# Pharmacodynamic Gene Testing in Prader-Willi Syndrome

**DOI:** 10.3389/fgene.2020.579609

**Published:** 2020-11-20

**Authors:** Janice Forster, Jessica Duis, Merlin G. Butler

**Affiliations:** ^1^Pittsburgh Partnership, Pittsburgh, PA, United States; ^2^Section of Genetics and Inherited Metabolic Disease, Department of Pediatrics, Children’s Hospital Colorado, University of Colorado Anschutz Medical Campus, Aurora, CO, United States; ^3^Division of Research and Genetics, Department of Psychiatry and Behavioral Sciences and Pediatrics, University of Kansas Medical Center, Kansas City, KS, United States

**Keywords:** genetic testing, medication management, imprinting, Prader-Willi, pharmacogenetics

## Abstract

Prader-Willi syndrome (PWS) is a rare genetic disorder with a complex neurobehavioral phenotype associated with considerable psychiatric co-morbidity. This clinical case series, for the first time, describes the distribution and frequency of polymorphisms of pharmacodynamic genes (serotonin transporter, serotonin 2A and 2C receptors, catechol-*o*-methyltransferase, adrenergic receptor 2A, methylene tetrahydrofolate reductase, and human leucocytic antigens) across the two major molecular classes of PWS in a cohort of 33 referred patients who met medical criteria for testing. When results were pooled across PWS genetic subtypes, genotypic and allelic frequencies did not differ from normative population data. However, when the genetic subtype of PWS was examined, there were differences observed across all genes tested that may affect response to psychotropic medication. Due to small sample size, no statistical significance was found, but results suggest that pharmacodynamic gene testing should be considered before initiating pharmacotherapy in PWS. Larger scale studies are warranted.

## Introduction

Pharmacogenomics is the study of how structural gene changes determine the function, regulation and production of gene products that affect the body’s response to medication. Pharmacodynamic testing involves the assessment of genes that code for neurotransmitter receptors and transporters, antigens, and enzymes that have an impact on drug activity and response, usually in the brain, as a function of an individual’s DNA pattern. Pharmacodynamic phenotypes may explain why certain classes of medications are not as effective in some people, beyond that predicted by the knowledge of drug pharmacokinetics and cytochrome P450 genes. Also, pharmacodynamic phenotypes may inform the risk of occurrence of side effects. Clinicians who are informed about the therapeutic value of pharmacodynamic testing appreciate and utilize this knowledge to evaluate potential side effects and variability of response to certain classes of psychotropic medication.

Using psychotropic medication to manage the multifaceted symptoms associated with Prader-Willi syndrome (PWS) requires a knowledge of evidence-based science, clinical expertise, and risk for potential side effects. PWS is a rare, complex, neurodevelopmental disorder best known for hypothalamic obesity, neuroendocrine disturbances, and psychiatric co-morbidity. PWS is an imprinting disorder resulting from absent paternal expression of the genes located in the chromosome 15q11-q13 region. The features of PWS include infantile hypotonia, a poor suck with feeding difficulties, global developmental delay, hypogenitalism/hypogonadism and growth failure due to multiple hormone deficiencies (Butler et al., 2006). Short stature with small hands and feet are common findings, and food seeking with hyperphagia develops in early childhood leading to morbid obesity, if not controlled by dietary and environmental restriction and mandatory exercise. Disorders of sleep (central and obstructive sleep apnea) and wakefulness (excessive daytime sleepiness) are common. Mild intellectual disability, learning difficulties, and behavioral concerns are noted. Temper tantrums, stubbornness, emotional outbursts, and skin picking may be present along with excessively rigid and repetitive behavior that may appear obsessive-compulsive like. Mood disorder, psychosis, and symptoms of Autism spectrum disorder are more likely to correlate with specific PWS genetic subtypes (Butler et al., 2004, 2006; Weisensel et al., 2015, 2016; Manzardo et al., 2018). A positive DNA methylation test confirms the diagnosis of PWS in 99% of cases, but additional testing is required to determine the genetic subtype. The 15q11-q13 deletion (DEL) occurs in 60% of cases, maternal disomy 15 (UPD), in which one inherits both chromosome 15 s from the mother, occurs in 35% of cases, and the remaining persons have defects in the imprinting center that controls the activity of the imprinted genes in the 15q11-q13 region (Butler et al., 2019). The frequency of maternal disomy 15 appears to be increasing among older mothers and those who have had *in vitro* fertilization (Butler et al., 2019; Hattori et al., 2019).

The spectrum of clinical findings in PWS presents challenges for families, caregivers and providers alike to develop and implement various management strategies (Duis et al., 2019). Clinical care is most effectively directed by a multidisciplinary team that includes clinical geneticists, endocrinologists, dietitians, exercise physiologists, gastroenterologists, orthopedic specialists, and primary care physicians. The primary goal of clinical care is to manage weight gain, monitor growth, and treat associated comorbid conditions such as hormone deficiencies, scoliosis, gut mobility issues, sleep apnea, and skin picking. Ancillary disciplines of speech and language therapy, physical therapy, occupational therapy, and special educators are essential toward the achievement of developmental and vocational goals. Mental health experts manage behavioral symptoms through modified delivery of behavioral analysis techniques and cognitive behavior therapy.

Psychiatric evaluation elucidates the etiology of symptoms, including diagnosing co-morbid psychiatric disorders, and results in a multidimensional treatment plan that involves environmental modification, enhancing coping skills, behavior management and use of psychotropic medication, hopefully in that order. Too often psychotropic medications are used for crisis intervention, and the evidence-base for use of psychotropic medication to manage aspects of the behavioral phenotype is lacking. Psychotropic medication selection and dosage determination are important for optimal care. Clinical experience suggests that people with PWS as a group appear to be more sensitive to psychotropic medications, requiring lower than typical doses for clinical response. But there are individual variations, and this is a focus of investigation in this report.

In 2006 the National Institutes of Health (NIH) PWS Rare Disease Registry began recruiting persons with PWS for the largest naturalistic longitudinal study examining factors related to the cause, manifestations and treatment of this rare genetic disorder (Butler et al., 2018). Of the 355 registrants in the NIH Rare Disease PWS Registry, 265 of them received 483 psychotropic medications listed in Table 1. The frequency of use by medication class is found in Table 2.

**TABLE 1:** Number, class and name of psychotropic medications prescribed to 265 PWS registrants in the National Institutes of Health Rare Disease Consortium for Prader-Willi Syndrome.

**Antidepressant/Anti-anxiety**	***N***	**Antipsychotic/Mood stabilizer**	***N***	**Anticonvulsant/Mood stabilizer**	***N***	**Stimulants/Non-stimulants**	****N****
**SSRI**	**143**	**SGA**	**90**	**Anticonvulsant**	**76**	**Methyphenidate**	**36**
Fluoxetine	58	Risperidone	37	Topiramate	32	Ritalin*	21
Sertraline	37	Aripiprazole	26	Valproic acid	17	Concerta*	7
Citalopram	18	Quetiapine	15	Phenobarbital	7	Focalin*	6
Escitalopram	15	Ziprasidone	8	Carbamazepine	2	Metadate*	1
Paroxetine	12	Paliperidone	2	Oxcarbazepine	4	Daytrana*	1
Fluvoxamine	3	Olanzapine	1	R-etiracetam	4	**Amphetamine**	**11**
**SNRI**	**8**	**FGA**	**10**	Lamotrigine	4	Adderall*	5
Nefazodone	1	Haloperidol	3	Gabapentin	2	Dexedrine*	5
Venlafaxine	4	Loxapine	3	Tiagabine	2	Vyvanse*	1
Desvenlafaxine	1	Thioridazine	3	Zonisamide	1	**Nonstimulant**	**39**
Imipramine	1	Chlorpromazine	1	Phenytoin	1	Atomoxetine	5
Clomipramine	1			**Mood stabilizer**	**4**	Modafanil	34
**Other**				Lithium	4		
Bupropion	12						
Trazadone	1						
**Anti-anxiety**	**37**						
Benzodiazepine	18						
Clonidine	8						
Guanfacine	3						
Buspirone	4						
Hydroxyzine	4						

SSRI, selective serotonin reuptake inhibitor; SNRI, serotonin norepinephrine reuptake inhibitor; SGA, second generation antipsychotics; FGA, first generation antipsychotics. *Brand name used. The bolded numbers in the table are subtotals for medication classes.

**TABLE 2:** Percentage of 265 persons from the NIH PWS registry receiving psychotropic medication by class.

**Medication class**	**Frequency of use %**
SSRI	54.0
SNRI + Other	7.9
Anti-anxiety	13.9
Atypical antipsychotics (SGA)	33.9
Typical antipsychotics (FGA)	3.8
Anticonvulsant/mood stabilizer	30.2
Stimulant	17.7
Non-stimulant	14.7

*SSRI, selective serotonin reuptake inhibitor; NSRI, non-selective serotonin reuptake inhibitor; SGA, second generation antipsychotics; FGA, first generation antipsychotics.*

The data from the NIH PWS Registry indicates that over the past 10 years, there has been a downward trend in the age that children with PWS first receive treatment with selective serotonin reuptake inhibitors (SSRIs). Nearly 50% of children age 5–12 year were receiving SSRIs, and this increased to 70% in the 12–21 years age group, often together with an atypical antipsychotic medication (Forster, 2019). Polypharmacy leads to drug-drug interactions, a significant problem contributing to medical and psychiatric co-morbidity (Olashore and Rukewe, 2017). Further, except for treatment of co-morbidities or specific augmentation strategies, the evidence base for using multiple medications simultaneously is lacking (Mrazek, 2010a). Therefore, there is a need for an informed and individualized approach to dose selection and medication management (Blasco-Fontecilla, 2019).

This clinical case series, for the first time in PWS, describes the clinical significance of the most commonly studied pharmacodynamic genes and their polymorphisms: serotonin transporter (SLC6A4), serotonin 2A receptor (HRT2A), serotonin 2C receptor (HRT2C), catechol-*o*-methyltransferase (COMT), adrenergic receptor 2A (ADRA2A), methylene tetrahydrofolate reductase (MTHFR), and human leucocytic antigens (HLA-A and B). None of the pharmacodynamic genes tested is located in the PWS chromosome 15q11-q13 region, which is deleted or not expressed in the majority of patients with PWS. To date, only serotonin 2C receptor activity and serotonin transporter activity have been identified as being affected in animal models and humans. There are epigenetic effects on the editing of serotonin 2C receptor believed to be caused by base pair complementarity with one of the sno-RNAs in the critical region, specifically *SNORD115* (Kishore and Stamm, 2006). However, the loss of *SNORD115* in animal models does not appear to be sufficient to cause the abnormal editing of HTR2C (Hebras et al., 2020). Falaleeva et al. (2015) has proposed a mutual interaction between SNORDs, where *SNORD116* is necessary to facilitate alternative splicing of RNAs by *SNORD115*, and *SNORD115* modulates the expression of genes by *SNORD116*, although none of those appear to be directly related to the pharmacodynamic genes in this case report (Falaleeva et al., 2015). Deficiency of *Necdin*, one of the imprinted genes not expressed in PWS, is related to an increase in serotonin transporter activity and altered neuroarchitecture of the serotonergic system in the *Necdin* knock-out mouse, specifically affecting the central respiratory mechanisms in the medulla (Zanella et al., 2008; Matarazzo et al., 2017). Also, decreased serotonin transporter has been identified in the brain stem of patients with PWS UPD vs. DEL using single photon emission tomography (SPECT) (Krishnadas et al., 2018). Similar imaging studies have noted a reduction in SERT in the brain stem and other brain regions of adults with depressive illness vs healthy controls (Kambeitz and Howes, 2015). This is relevant to PWS given the greater prevalence of affective psychosis among UPD than DEL (Singh et al., 2019a). Finally, MTHFR TT polymorphism, which is the low activity polymorphism, was higher in mothers who had a child with Angelman syndrome due to a maternal imprinting defect, a deficiency of the methylation process, but data from fathers did not reach statistical significance (Zogel et al., 2006). It should be noted that the American College of Medical Genetics does not endorse MTHFR testing because of population heterogeneity and minimal data for clinical utility in predicting risk for coronary artery disease or thromboembolism. The clinical utility of MTHFR polymorphism studies in psychiatry continues to be examined (Wan et al., 2018).

For the first time, the frequency of pharmacodynamic gene polymorphisms in this cohort of clinically referred patients with PWS is presented and compared with normative population data when possible. The capacity to use this knowledge to anticipate treatment efficacy and/or vulnerability to adverse effects of pharmacotherapy will be addressed. Finally, the potential for interactions between psychodynamic genes to affect psychiatric co-morbidity in genetic subtypes of PWS is discussed.

## Materials and Methods

In this clinical case series, 33 patients with genetically confirmed PWS were evaluated at one of three geographically distinct clinical centers in the United States (University of Kansas Medical Center, Vanderbilt University Medical Center and Pittsburgh Partnership, PA, United States). Pharmacogenomic testing was ordered as part of the clinical evaluation by the authors who are physicians treating patients with medications prescribed for behavioral or psychiatric problems. All patients in this clinically referred cohort met medical necessity criteria for testing. After a discussion with the physician about pharmacogenetics and its use in medication management, the guardians consented for genetic testing by signing order forms provided and approved by the three commercially licensed and CLIA accredited laboratories: Genesight (Mason, OH, United States), Genelex (Seattle, Washington), and Genomind (King of Prussia, PA, United States). Buccal cells were collected from a total of 33 patients in the clinical setting and sent to the laboratories of Genesight (*n* = 27), Genelex (*n* = 5), and Genomind (*n* = 1) for DNA extraction and genotyping. The results were received by each clinician, deidentified with respect to age, gender, race and ethnicity, and added to the collective data set. Information about clinical treatment and response to psychotropic medication(s) in our PWS cohort was not included, although clinical necessity criteria for testing requires treatment failure due to inefficacy, treatment emergent side effects, or co-morbid conditions requiring multiple medications. Only the PWS genetic subtype was identified in the population. There were 14 persons with the 15q11-q13 deletion (DEL) and 14 persons with uniparental maternal disomy 15 (UPD). Genetic subtype was unknown in 5 persons whose diagnosis of PWS was confirmed previously with DNA methylation. The most commonly studied pharmacodynamic genes and their polymorphisms were analyzed: serotonin transporter (*SLC6A4*), serotonin 2A receptor (*HTR2A*), serotonin 2C receptor (*HTR2C*), *COMT*, adrenergic receptor 2A (*ADRA2A*), *MTHFR*, and human leucocytic antigens (*HLA-A* and *HLA-B*). Results were collated across PWS genetic subtypes, and a third category comprised of pooled data (DEL + UPD + PWS unspecified) was designated as “ALL.” Genotype and allele frequencies were calculated and compared with normative population data. The frequency of pharmacodynamic gene polymorphisms has been based upon large population studies with ethno-geographic specificity. When possible, the data derived from our cohort of persons with PWS was compared to genotypic and allelic frequencies from populations designated as North American, European American, European, White American, or US Caucasian by chi-square test. The phenotype of each pharmacodynamic gene was inferred from the genotype. For example, it is well known that the s allele of the serotonin transporter promoter region has been associated with 50% less transcription than the L allele, so the homozygous form is designated as low activity. However, it is also acknowledged that there are other factors that may determine transcriptional activity (Mendlewicz et al., 2004).

Ethical review and approval were not required for this report. This article is a scholarly report of a clinical case series of patients with PWS who received pharmacogenomic testing as part of medical care at one of three specialty programs across the United States. This report will both inform and hopefully improve the quality of care of patients with PWS. The clinical care described in this article was not part of a research project. Prior to the collation and analysis of data, all pertinent private or protected health information about each patient was eliminated, except for the genetic subtype of PWS and the pharmacodynamic genotypes. Further, the results presented in this paper have been deidentified, will not affect the patient’s clinical care, nor do they have the potential to cause the patient any harm. Therefore, IRB approval was not obtained for ethics oversight.

## Results

This is the first attempt to ascertain the frequency of polymorphisms of pharmacodynamic genes in persons with PWS. From the frequency data presented in the combined cohort of “ALL” PWS, polymorphisms of pharmacodynamic genes tested were similar to normative population data. However, differences were observed between DEL and UPD genetic subtypes. In summary, those with UPD displayed an increased frequency of the L allele of the serotonin transporter and an increased frequency of VAL/VAL polymorphism of *COMT* gene. Those with DEL displayed increased frequency of HRT2A polymorphisms associated with risk of autonomic and respiratory dysfunction in infancy and sleep apnea and metabolic syndrome with age. Among patients of both subtypes, there was an increased frequency of serotonin 2C polymorphism resulting in potential risk for side effects associated with pharmacotherapy with SSRIs and atypical neuroleptics. There were genetic subtype differences for the ADRA2A gene suggesting increased risk of side effects in DEL and decreased efficacy in UPD during treatment with alpha-adrenergic agonists. MTHFR results indicate that both PWS genetic subtypes have an increased frequency of diminished function alleles, as well as increased frequency of compound genotypes associated with potential risk of developing psychosis. In summary, when analyzed by genetic subtype, there are pharmacodynamic gene differences in this referred population that may contribute to response and/or the emergence of adverse effects with treatment. Although the above findings showed differences, they were not significantly different by chi-square test (*p* < 0.05) due to small sample size. The complete results of the pharmacodynamic genes tested (*SLC6A4, HTR2A, HTRT2C, ADRA2A, COMT*, and *MTHFR*) in our referred cohort clustered by genetic subtype and testing lab are found in Table 3.

**TABLE 3:** Selected results of pharmacodynamic genotypes and phenotypes among the referred cohort of patients with PWS assorted by genetic subtype and pharmacogenomic testing lab.

	***SLC6A4***	***HTR2A***			***HTR2C***	***ADRA2A***	***COMT***		***MTHFR***	
**Lab**		**1438G > A**	**−998G > A**	**614-2211T > C**	**−759C > T**	**−1291G > C**	**VAL^158^MET**	**472G > A**	**677C > T**	**1298A > C**
	**“PWS”**									
GS	L/s	GA t								
GS	s/s	GA i				CC d	VAL/MET i		CT i	
GS	L/L	GG r				GC t	VAL/MET i			
GS	L/L	AA d				CC d	MET/MET d		CC t	
GS	s/s	GA t				GC t	VAL/MET i		CT i	
	**DEL**									
GL			GA i	CC d	CC r	CC d		AA d	CT i	AC i
GS	L/s	GG r				GC t	VAL/MET i			
GL			GG r	CC d	CC r	GC t		AA d	CT i	CC d
GL			AA d	TC t	CC r	GC t		AA d	CT i	AA t
GS	s/s	GG r				CC d	VAL/MET i			
GS	L/s	GA t				GC t	VAL/MET i			
GS	L/s	GA t				GC t	MET/MET d			
GS	s/s	AA d				CC d	MET/MET d		CC t	
GL	LA/sA		AA d	CC d	CC r	GC t		AA d	TT d	AA t
GS	s/s	GA t				GC t	VAL/MET i			
GS	L/L	AA d				CC d	VAL/MET i			
GS	L/L	GG r				GG t	VAL/VAL h			
GS	s/s	GA t				CC d	VAL/VAL h			
GS	L/s	GA t				GC t	MET/MET d			
	**UPD**									
GL			GA i	TC t	CC r	GC t		GA i	CC t	AC i
GS	L/L	GA t				CC d	VAL/VAL h			
GS	L/s	GA t				GC t	MET/MET d		CC t	
GS	L/s	GA i				GC t	VAL/VAL h		CT i	
GS	L/s	GA i				CC d	VAL/VAL h		CT i	
GS	L/s	GA t				CC d	VAL/MET i		CT i	
GS	L/L	GA t				CC d	VAL/MET i		CC t	
GS	L/L	GA i				GC t	VAL/VAL h		CC t	
GS	L/s	GA t				CC d	VAL/MET i			
GM	L/L	GA t								
GS	L/L	GG r				GC t	MET/MET d			
GS	L/L	GA t				CC d	VAL/VAL h			
GS	L/L	GG r				CC d	VAL/VAL h			
GS	L/s	GA t				CC d	VAL/MET I			

*Lab: GS, GeneSight; GL, Genelex; GM, Genomind. PWS Genetics: DEL, Deletion; UPD, Uniparental disomy. “PWS” – Methylation positive, subtype unspecified. Phenotypes: d, decreased activity; i, intermediate activity; t, typical activity; h, high activity; r, at risk.*

### Serotonin Transporter

One of the best studied pharmacodynamic genes is the serotonin transporter (5-HTT, SERT, or SLC6A4) that has both short and long polymorphisms of the gene promoter allele. The SERT gene is located at Ch17q11.1-q12. The serotonin transporter shuttles through the synaptic cleft from the serotonergic neuron to the post-synaptic receptor, collecting and recycling unbound serotonin. The homozygous form of the short allele (s/s) is associated with reduced efficiency of transcription, lower gene expression, and decreased serotonin binding affinity (Lesch et al., 1996). Both human and animal studies have shown that this genotype is associated with increased stress sensitivity to adverse life events, although a positive outcome can be expected with environmental support (Kumsta et al., 2010). The ss genotype is associated with anxious, neurotic and somatic personality styles (Caspi et al., 2003), not unlike some of the personality characteristics seen in PWS. The s allele appears to modulate the impact of environmental stress on depression (Daws and Gould, 2011). Studies have demonstrated an additive effect of the number of s alleles contributing to low emotional resilience and distress intolerance (Stein et al., 2009; Amstadter et al., 2012). Schepers and Markus (2017a, b) found that BMI was positively correlated with a ruminative cognitive style among s allele carriers, and that in this group, stress increased attention bias for high calorie, savory foods identified by an eye-tracking task. The homozygous form of the long allele (L/L) is associated with high gene expression, and this genotype confers stress resilience (Stein et al., 2009). Medications of the selective serotonin reuptake inhibitor (SSRI) type bind with variable affinity to the transporter, displacing serotonin into the cleft for increased availability at the post-synaptic receptor. This is the mechanism for producing the antidepressant response from these medications. The ss genotype is associated with decreased response to SSRI medications, and the LL genotype is associated with increased response. Although the genotype is subject to Mendelian inheritance, there is variability of gene frequency across ethno-geographic groups as discussed by Mrazek (2010b). This case series infers the phenotype from the genotype, although there are epigenetic factors that ultimately determine gene expression such as nutrition, stress, immune activation, age, and pharmacological effects as discussed by Daws and Gould (2011).

**FIGURE 1:**
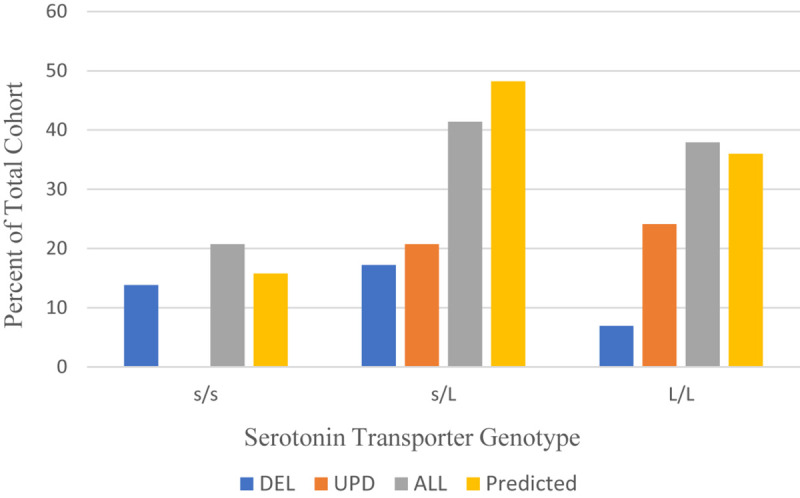
Distribution of serotonin transporter genotypes by PWS genetic subtype ALL (*n* = 29), DEL (*n* = 11), UPD (*n* = 13) compared to a predicted Caucasian American population (*n* = 114) (Poland et al., 2013). DEL, deletion; UPD, uniparental disomy; ALL, combined cohort of PWS.

The distribution of serotonin transporter genotype among the 29 patients with PWS in our cohort compared to the Caucasian American population is shown in Figure 1. The alleles in the combined cohort “ALL” show some skewing toward the L/L genotype comparable to the predicted cohort.

Table 4 shows the allelic frequencies for the combined cohort of PWS (ALL), which are 59% for the L allele and 41% for the s allele. These frequencies compare favorably with the allelic frequencies reported in the Caucasian American population, which are 60% for the L allele and 40% for the s allele (Poland et al., 2013). However, the data is different when the genetic subtype of PWS is considered. For the UPD subtype, the distribution is skewed toward the L/L genotype, and no one with UPD had the s/s genotype. The frequency of the L allele among our UPD cohort is 76.9%. This is higher than the expected 60% ascertained for the Caucasian American population (Poland et al., 2013). This finding may indicate increased sensitivity to SSRIs and greater possibility of mood activation. Results for the deletion cohort are skewed toward the s/s genotype. The frequency of s allele is 59.1% among the deletion subgroup, higher than the 40% expected for the Caucasian American population (Poland et al., 2013). This finding is consistent with what might be expected for persons with PWS, who show traits of stress sensitivity, neuroticism and somatization and have a better outcome in a structured, socially supported environment.

**TABLE 4:** Distribution of serotonin transporter genotype and allele frequencies among PWS DEL, UPD and ALL cohort (*n* = 29) compared to a predicted Caucasian American population (*n* = 114); both number and within group (%) are reported.

**Cohort**	***N***	**s/s (%)**	**s/L(%)**	**L/L(%)**	**s Allele**	**L Allele**
Caucasian American*	114	18(15.8)	55(48.2)	41(36)	0.40	0.60
Current: ALL PWS	29	6(20.7)	12(41.4)	11(37.9)	0.41	0.59
DEL subtype	11	4(36.4)	5(45.5)	2(18.2)	0.59	0.41
UPD subtype	13	0	6(46.2)	7(53.8)	0.23	0.77

*Poland et al., 2013.

### Serotonin Receptors

**FIGURE 2:**
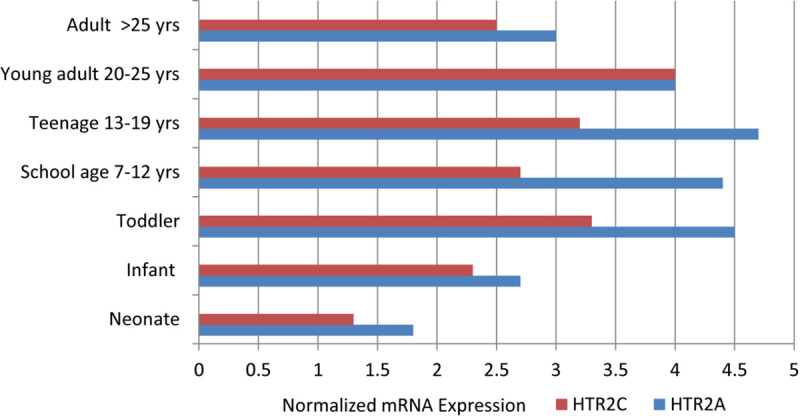
Normalized mRNA expression of serotonin receptors in human prefrontal cortex obtained from post-mortem cases of different ages, as adapted from Lambe et al. (2011).

Serotonin receptors 2A (*HTR2A*) and 2C (*HTR2C*) are most relevant to understanding the action and efficacy of antidepressant medication. They are G-protein coupled, post-synaptic receptors with expression developmentally regulated in the prefrontal cortex (PFC) where two serotonin receptors appear have opposing effects. Serotonin 2A receptors are located on excitatory glutamate neurons while HTR2C are located on inhibitory GABA interneurons. Lambe et al. (2011) studied gene expression (mRNA) from *HTR2A* and *HTR2C* in the PFC of post-mortem brains sampled at various ages across the life cycle (Figure 2).

Expression of the mRNA from *HTR2A*, the excitatory serotonin receptor, increases from infancy through the toddler and school age years, peaks during adolescence, and then declines in the young adult years until reaching adulthood levels. Expression of the mRNA from *HTR2C*, the inhibitory serotonin receptor, follows a similar pattern of upregulation at a level two thirds of *HTR2A* expression until parity is achieved in the young adult and later adult years. The greatest discrepancy in expression occurs in adolescence. From this mRNA data in the human prefrontal cortex, it appears that the activating form of *HTR2A* expression predominates from the school age years through adolescence. It is well known that the phenomenon of mood and behavioral activation associated with the use of SSRIs is more likely to occur in younger individuals and adolescents (Luft et al., 2018). This is the reason for the black box warning about suicide potential among children and adolescents started on SSRIs and some atypical antipsychotics. Further, Pandey et al. (2002) found an increase in the serotonin 2A receptors in the PFC of post-mortem brains of adolescents who completed suicide, and Schmauss (2003) found abnormal editing of the serotonin 2C receptor in the PFC of depressed suicide victims.

This information is particularly relevant to persons with PWS. Located in the imprinted region of chromosome 15q11-q13 is a small nucleolar RNA (HBII-52 or SNORD115) that is required for proper editing of the serotonin 2C receptor (Kishore and Stamm, 2006). The clinical implication for PWS is that the serotonin 2C receptor is produced, but its overall efficacy is diminished due to improper editing. Therefore, it is likely that persons with PWS have a serotonin 2C receptor with deficient function. With a typical expression of *HTR2A* throughout development and lower functional expression of *HTR2C* in PWS leading to less inhibitory effect, there is greater potential for clinical response to lower doses of SSRIs as well as adverse effects of mood activation at typical doses (Durette et al., 2012; Gourash et al., 2015).

#### Serotonin 2A Receptor

**FIGURE 3:**
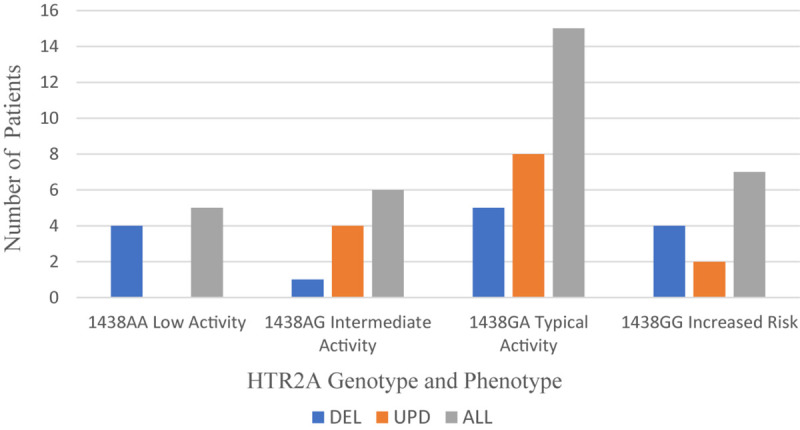
Frequency of HTR2A genotype and phenotype per PWS genetic subtype cohort: DEL (*n* = 14), UPD (*n* = 14), and ALL (*n* = 33). DEL, deletion; UPD, uniparental disomy; ALL, combined cohort of PWS.

The serotonin receptor 2A (*HTR2A*) gene is located on chromosome 13q14-21. Only the rs6311 polymorphism in the promoter (−1438G > A) of *HTR2A* is tested on the Genesight panel. The −998G > A polymorphism on the Genelex panel is the same as −1438G > A. The phenotypic activity of the −1438GA allele has been reported as typical or intermediate by GeneSight and Genelex. The frequencies of the genotypes and phenotypes reported in the PWS genetic subgroups are displayed in Figure 3.

Across the total PWS cohort, the typical phenotype of −1438G > A is expressed more frequently (45.5%) than the intermediate phenotype (18.26%). The −1438AA genotype, a low activity phenotype, is reported in 15.2% of the total cohort. In several meta-analyses, the AA genotype carries the greatest risk for obstructive sleep apnea and hypopnea (Zhao et al., 2013; Xu et al., 2014). The -1438GG genotype occurs in 21.2% of the total cohort, and this phenotype carries the greatest risk for increased waist circumference associated with metabolic syndrome and hypertension (Halder et al., 2007).

Among 286 individuals of European ancestry, the prevalence of −1438G > A polymorphisms was 50 AA (17.5%), 144 GA (50.3%), and 92 GG (32.2%); the frequency of the G allele was 0.57 and the frequency of the A allele was 0.43 (Gorwood et al., 2002). Among the total PWS cohort, the frequency of the G allele was 0.53, and the frequency of A allele was 0.47, similar to that reported by Gorwood et al. (2002).

Table 5 displays the genotypic frequencies among the PWS genetic subtypes and compares them to the typical European population described above. There was a more normal distribution of -1438G > A gene polymorphisms in the DEL subtype: AA (28.6%), GA (42.9%), and GG (28.6%) where the frequency of both A and G alleles was 0.50. In the UPD subtype, the distribution of polymorphisms was skewed: AA (0), GA (86.7%), and GG (14.3%) with the frequency of the G allele being 0.57 and the A allele 0.43, remarkably similar to that reported by Gorwood et al. (2002) in the typical European population. So, compared to UPD, the deletion subtype in our cohort appears to be at greater risk for adverse effects of SSRIs, sleep apnea and metabolic syndrome.

**TABLE 5:** Distribution of HTR2A genotypes and allele frequencies among PWS and European cohorts; both number and within group (%) are reported.

**Cohort**	**N**	**AA(%)**	**GA(%)**	**GG(%)**	**A**	**G**
European*	286	50(17.5)	144(50.3)	92 (32.2)	0.43	0.57
Current: ALL PWS	33	5(15.2)	21(63.6)	7(21.2)	0.47	0.53
DEL subtype	14	4(28.6)	6(42.9)	4(28.6)	0.50	0.50
UPD subtype	14	0	12(86.7)	2(14.3)	0.43	0.57

*Gorwood et al., 2002.

In addition to the *HTR2A* -998G > A polymorphism, the Genelex panel also tests for the 614-2211T > C allele, also known as rs7997012 or −1178G > A. In the STAR^∗^D study, people with depression who had the adenosine substitution (AA) were more likely to respond to citalopram than those who had the GA or GG genotype (McMahon et al., 2006). Of the five patients tested using the Genelex panel in our case series, three had the low activity phenotype and two had typical activity.

In addition to phenotypic differences in functional activity determined by the polymorphism, binding affinity varies across drugs. Among the atypical antipsychotic medications, risperidone and ziprasidone have the highest affinity for HTR2A followed by olanzapine and aripiprazole. Increased risk for movement disorder, such as tardive dyskinesia, which is an adverse effect associated with the use of antipsychotic medications, is increased among those with the G allele of −1438G > A (rs6311) and the C allele of 102T/C (rs6313) (Lattuada et al., 2004). These two polymorphisms are in strong linkage disequilibrium with rs1928040, another polymorphism of *HTR2A* associated with the orofacial type of tardive dyskinesia particularly among females (Pozhidaev et al., 2020).

The promoter region of *HTR2A* is subject to epigenetic modification by methylation during fetal development, and these effects have been associated with neurobehavioral problems during infancy, psychiatric disorders in young adulthood, and chronic fatigue syndrome in adults as discussed by Paquette and Marsit (2014). Stress has an ongoing role in epigenetic regulation of *HTR2A*, and there are both glucocorticoid as well as cortisol binding sites in the promoter region (Paquette and Marsit, 2014).

#### Serotonin Receptor 2C

The Serotonin 2C receptor gene (*HTR2C*) is located on the X chromosome (Xq24). So males, who by definition have only one X chromosome, are maternally hemizygous for the *HTR2C* gene alleles. The serotonin 2C receptor is a site of action of most antidepressants and atypical antipsychotic medications. All these agents have variable affinity for the receptor. Polymorphisms of the *HTR2C* are associated with increased risk of side effects of weight gain and movement disorder with the use of atypical antipsychotics (MacNeil and Müller, 2016). Only Genelex tests for polymorphisms of the serotonin 2C receptor gene, so the data from our PWS cohort is limited. Of the five cases receiving Genelex testing of the *HTR2C* -759 C > T polymorphism, all of them had the CC phenotype suggesting greater risk of side effects of weight gain and movement disorder associated with the use of atypical antipsychotic medications. As previously described, the function of the serotonin 2C receptor is likely to be abnormal in PWS.

### Alpha-2A Adrenergic Receptor

The alpha-2A adrenergic receptor is a G-protein coupled receptor that primarily provides presynaptic feedback inhibition on norepinephrine (NE) release by central and peripheral sympathetic neurons. This results in reduced peripheral vasoconstriction and blood pressure. The gene is located at Ch10q25.2. The -1291C/G promoter polymorphism in *ADRA2A* has been associated with vascular reactivity to stress, and vasoconstriction increases linearly with the number of copies of the G allele (Flordellis et al., 2004). Specifically, there is an association between this genetic variation and cardiovascular reactivity in young African Americans (Kelsey et al., 2012). Also, the adrenergic receptor 2A modulates response to methylphenidate and alpha-adrenergic agonists. There is a linear relationship between the number of G substitutions in the −1291C/G promoter polymorphism and the response to methylphenidate targeting inattentive symptoms in ADHD (Schmitz et al., 2006; Polanczyk et al., 2007; Cheon et al., 2009). However, more recent studies have implicated a more robust pharmacodynamic association between a diagnosis of ADHD and serotonin transporter, dopamine transporter, dopamine receptor, *COMT* and other pertinent genes (McGough et al., 2009; Joensen et al., 2017).

ADRA2A (−1291CG) and other adrenergic receptor SNPs are also involved in metabolism and central obesity (Halder et al., 2007). Alpha-2A adrenergic receptors are located in pancreatic islet β-cells, and there is an association between stress and hyperglycemia associated with stress, especially among Caucasians (Adefurin et al., 2016). Insulin activates *ADRA2A*, and expression of *ADRA2A* inhibits lipolysis, contributing to weight gain (Stich et al., 1999, 2003). Garenc et al. (2002) found an association between some SNPs of *ADRA2A* with the accumulation of abdominal fat unrelated to overall body mass among African Americans. Although *ADRA2A* alone has not been associated with obesity, there is an interactive effect with other SNPs (Lima et al., 2007). Some atypical antipsychotics such as risperidone have alpha-adrenergic agonist effects. Given that hyperglycemia and an accumulation of abdominal fat is associated with adrenergic agonist effects, it is possible that the weight gain and metabolic syndrome occurring during treatment with second generation antipsychotics may be related, in part, to their adrenergic effects (Riordan et al., 2011).

**FIGURE 4:**
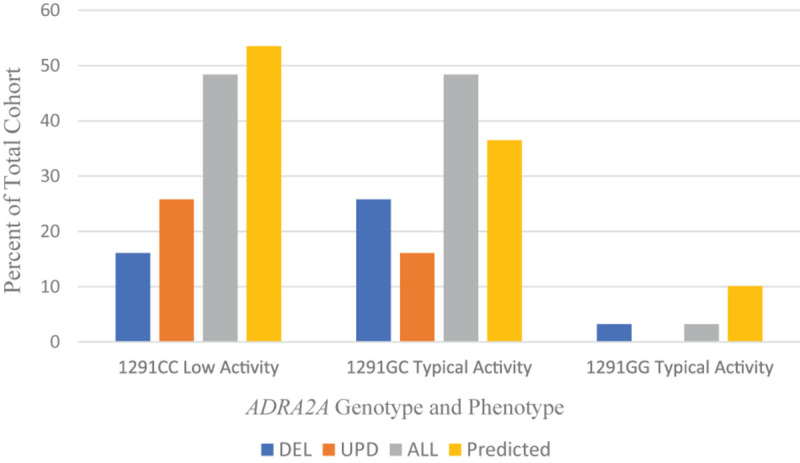
Frequencies of 1291C > G *ADRA2A* genotype and phenotype among PWS genetic subtypes DEL, UPD, and ALL (*n* = 31) compared to a predicted cohort of White Americans (*n* = 232) (Lima et al., 2007). DEL, deletion; UPD, uniparental disomy; ALL, combined cohort of PWS.

Figure 4 shows the frequency of the -1291C/G promoter polymorphism in *ADRA2A* in our cohort of patients with PWS. Overall, the frequency of polymorphisms is almost evenly distributed between the low affinity and typical affinity phenotypes; there was only one person with the *ADRA2A*−1291GG allele, which also confers the typical affinity phenotype. When examining the results for the genetic subtypes of PWS, there is a tendency toward the typical affinity phenotype among the deletion group and the low affinity phenotype among the UPD group.

Population studies show a variation in the distribution of −1291C/G polymorphism with race and ethnicity, as reviewed by Karahalil et al. (2008). The genotype and allelic frequencies among White Americans (Lima et al., 2007) are compared to the data from our PWS cohort in Table 6.

**TABLE 6:** Distribution of 1291C > G *ADRA2A* genotypes and allele frequencies among PWS and White American cohorts; both number and within group (%) are reported.

**Cohort**	***N***	**CC(%)**	**CG(%)**	**GG(%)**	**C**	**G**
White Americans*	232	85(53.5)	58(36.5)	16(10.1)	0.72	0.28
Current: ALL PWS	31	15(48.4)	15(48.4)	1(3.2)	0.73	0.26
DEL subtype	14	5(35.7)	8(57.1)	1(7.1)	0.64	0.36
UPD subtype	13	8(61.5)	5(38.5)	0	0.81	0.19

*Lima et al., 2007.

The frequency of the G allele is increased among the DEL subgroup. An increased dose of G allele has been associated with increased vascular reactivity with stress and improved response to methylphenidate and alpha-adrenergic agonists. There is an increase in the C allele among the UPD subgroup that may contribute to lesser efficacy of alpha-adrenergic agonists in treatment trials. The alpha-2 adrenergic receptor agonist guanfacine has been used successfully in patients with PWS to target symptoms of aggression, skin picking, and ADHD (Singh et al., 2019b), but it was not effective in managing the severe psychopathology associated with PWS, which is usually found in UPD.

### Catechol-*O*-Methyltransferase

The COMT enzyme is responsible for clearing dopamine and other monoamine neurotransmitters from the synaptic cleft. *COMT* polymorphism is a major determinant of dopamine function in the prefrontal cortex, limbic system and reward centers of the brain (Schacht, 2016). Dopamine tone in the prefrontal cortex determines cognitive functions along a U-shaped continuum; either too much or too little dopamine can impair executive function (Meyer-Lindenberg and Weinberger, 2006). Bilder et al. (2004) described an inverse relationship between cortical and subcortical activation associated with *COMT* polymorphism, dopamine tone, and psychosis susceptibility. Further, Mier et al. (2010) performed a meta-analysis of fMRI studies that supported a *COMT* polymorphism-related bias for cognitive vs emotional processing as a function of activation of the prefrontal cortex. In another meta-analysis, Taylor (2018) reviewed the association between *COMT* polymorphisms and major psychiatric disorders, such as schizophrenia, schizoaffective disorder, bipolar disorder, depression, obsessive compulsive disorder and ADHD. Many studies have explored how specific behavioral attributes (endophenotypes), such as stress sensitivity, reward response, extraversion, neuroticism and aggression are related to *COMT* polymorphisms (Hoth et al., 2006; Collip et al., 2011; Lancaster et al., 2012; Lee and Prescott, 2014; Qayyum et al., 2015; Millenet et al., 2018). Some studies have focused on the association of age across development and *COMT* expression (Bowers et al., 2020).

The COMT gene is located on chromosome 22q11.21. The rs4680 or 472G > A gene variant is the most widely studied. It occurs as a VAL/MET polymorphism and appears in 3 combinations (VAL/VAL, VAL/MET, and MET/MET) depending on a substitution of methionine for valine. The frequency of occurrence of the MET allele differs across geographical ancestry as reviewed by Mrazek (2010b). Also, there are racial differences with African Americans displaying a decreased frequency of MET/MET polymorphism (Fiocco et al., 2010).

*COMT* (VAL/VAL) is associated with high enzyme activity resulting in lower dopamine levels in the synaptic cleft. This has been associated with greater cognitive flexibility and a positive response to environmental support, but a greater likelihood of distractibility; it predicts a typical response to psychostimulants (He et al., 2012).

*COMT* (MET/MET) is associated with low enzyme activity (as much as 2–4 times lower than VAL/VAL) and higher dopamine levels in the synapse (Chen et al., 2004) of neurons in the PFC. It is associated with reward focused behavior and response (Lancaster et al., 2012) as well as stress sensitivity (He et al., 2012; Boecker-Schlier et al., 2016). MET/MET carriers are more likely to respond to stressful life events occurring in childhood and adolescence (Crum et al., 2018). This endophenotype may result in stress sensitivity throughout life and may predispose to addiction (Lovallo et al., 2017; Buchanan and Lovallo, 2019). Collip et al. (2011) found that the persons with the MET/MET phenotype were more emotionally reactive to stress, displayed more negative affect, and were more likely to experience “momentary psychosis” (most often with delusional thinking) in response to a stress. However, in a stress-is-enhancing mindset manipulation, MET/MET carriers were more likely than VAL/VAL carriers to reappraise the effect of stress on their lives and respond with improved cognitive function and positive affect (Crum et al., 2018). Also, MET/MET carriers who have schizophrenia or schizoaffective disorder are more likely to respond to treatment with atypical antipsychotic medications affecting dopamine receptors (D2) resulting in a reduction in positive symptoms (Huang et al., 2016; Schacht, 2016).

**FIGURE 5:**
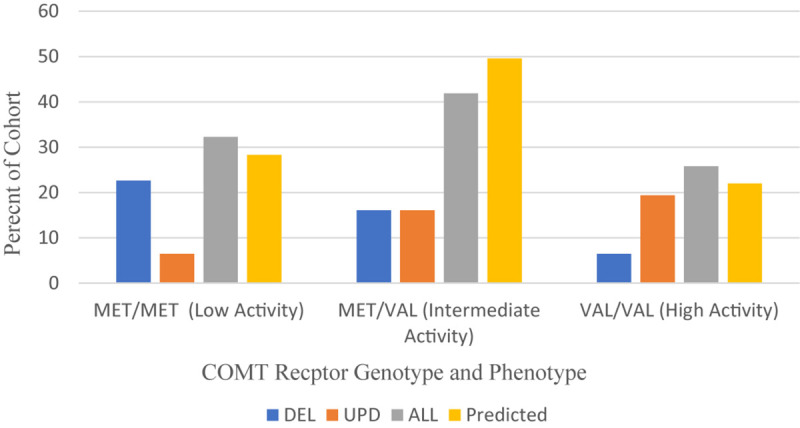
Frequency of COMT receptor phenotypes and genotypes among PWS genetic subtypes DEL, UPD, and ALL (*N* = 31) compared to a predicted Caucasian Cohort (*N* = 13,399) (Lee and Prescott, 2014). DEL, deletion; UPD, uniparental disomy; ALL, combined cohort of PWS.

Our cohort of 33 patients with PWS was sampled from clinics in the United States. The distribution of *COMT* polymorphisms is found in Figure 5 and compared to a normal Caucasian population (Lee and Prescott, 2014).

Overall, the MET/MET genotype was found in 32.3% of our collective cohort, which is higher than predicted (28.3%), and the VAL/VAL genotype was found in 25.8% of the collective cohort, also higher than predicted (22%) from a meta-analysis of studies yielding a normal Caucasian population of 13,399 people (Lee and Prescott, 2014). These data sets are comparable. When examining the results from the PWS genetic subtypes, however, the distribution is skewed toward MET/MET among those with DEL subtype and VAL/VAL among those with UPD.

Table 7 compares the *COMT* genotype and MET and VAL allelic frequencies of the PWS genetic subtypes with a normative Caucasian population. The frequency of MET allele among PWS with deletion is 0.68, which is higher than expected compared to the frequency among Caucasians, which is 0.53 (Lee and Prescott, 2014). The frequency of MET allele among PWS with UPD was 0.35, which is lower than Caucasian norms. Karayiorgou et al. (1997) looked at *COMT* polymorphisms among a large cohort of people with obsessive compulsive disorder (OCD) and found that among male subjects, the Met allele frequency was 0.68. The Type I deletion in PWS is more likely to be associated with obsessional thoughts and compulsive behaviors compared to Type II deletion or UPD (Manzardo et al., 2018). In the cognitive dimension, MET carriers have sustained dopamine activation in neural networks that promotes attention fixation and protect from distraction (Lee and Prescott, 2014). VAL carriers are better able to switch sets, update neural networks with new information, and extinguish patterns associated with rewards (Lee and Prescott, 2014). Further studies are required to ascertain the association between *COMT* polymorphisms and the unique neuropsychiatric phenotype associated with the PWS genetic subtypes.

**TABLE 7:** Comparison of COMT genotype and allele frequencies in PWS cohort (*n* = 31) with Caucasians (*N* = 13,399); both number and within group (%) are reported.

**Cohort**	***N***	**MET/MET**	**MET/VAL**	**VAL/VAL**	**MET**	**VAL**
Caucasians*	13,399	3,796 (28.3%)	6,650(49.6%)	2,953(22.0%)	0.53	0.47
Current: ALL PWS	31	10 (32.3%)	13 (41.9%)	8 (25.8%)	0.53	0.47
DEL subtype	14	7 (50.0%)	5 (35.7%)	2 (14.3%)	0.68	0.32
UPD subtype	13	2 (15.4%)	5 (38.5%)	6 (46%)	0.35	0.65

*Lee and Prescott, 2014.

### Methylene Tetrahydrofolate Reductase

Methylene tetrahydrofolate reductase is an enzyme that converts dietary folate into L-methyl folate, the building block of brain monoamine neurotransmitters and the catalyst of brain energy processes. This biochemical process is responsible for the methylation of homocysteine to produce methionine and eventually *S*-adenosylmethionine (SAMe). This epigenetic process of DNA methylation is the one-carbon metabolic pathway essential for DNA and neurotransmitter synthesis. When MTHFR function is impaired, abnormalities of the methylation cycle lead to oxidative stress (Fryar-Williams, 2016). As a result, serum folate may decrease, and serum homocysteine may increase. This contributes to a variety of illnesses such as cardiovascular disease, recurrent pregnancy loss, neural tube defects, cancer, leukemia, venous thrombosis and stroke, which require genetic counseling (Levin and Varga, 2016). *MTHFR* polymorphisms have been associated with Autism spectrum disorder (Rai, 2016), ADHD (Gokcen et al., 2011; Baykal et al., 2019), and migraine (Liu et al., 2019). Their association with neuropsychiatric disorders, such as depression, bipolar disorder and schizophrenia, has been well studied as reviewed by Gilbody et al. (2007), Peerbooms et al. (2011), Kevere et al. (2012), Hu et al. (2015), and Wan et al. (2018). Also, *MTHFR* polymorphisms have been associated with severity of illness, longer duration of symptoms, diminished response to medication as well as risk of side effects. In particular, the low activity *MTHFR* alleles have been found to contribute to symptoms of metabolic syndrome in patients receiving atypical neuroleptics (Ellingrod et al., 2012; Misiak et al., 2017). L-methyl folate has been accepted as a method to augment treatment of depression and bipolar disorder (Coppen and Bolander-Gouaille, 2005; Lewis et al., 2012; Baek et al., 2013).

The MTHFR gene is located on chromosome 1p36.22. The two most common single nucleotide polymorphisms are 677C > T and 1298A > C, both of which result in a proportional decrease in enzyme function across allelic frequencies. The 677C > T polymorphism alleles have been associated with a reduction in folate levels in erythrocytes (Molloy et al., 1997). Population studies in the USA have noted the association of the 677C > T polymorphism alleles (CC, CT, and TT) with race (Wilcken et al., 2003). Further, there is an ethno-geographical association with 677C > T polymorphism that varies across the world according to the distance from the equator (Wilcken et al., 2003; Castro et al., 2004; Mischoulon et al., 2012). When comparing results of *MTHFR* polymorphism studies race, ethnicity and geography should be considered.

Studies suggest a relationship between the *MTHFR* 677C > T mutation and increased susceptibility for depression (Lewis et al., 2006, 2012), and risk of depression has been found to increase with environmental stress and the dosage of T allele (Lok et al., 2013). Low serum folate, vitamin B12 and elevated serum homocysteine have variously been indicated (Bjelland et al., 2003). *MTHFR* polymorphisms have been reported to be associated with treatment resistant depression (Pan et al., 2017). Mischoulon et al. (2012) found that serum folate was negatively associated with treatment response in a study of adults with major depressive disorder (MDD). Supplementation with folate and vitamin B12 has been considered as an adjunctive treatment for depression (Coppen and Bolander-Gouaille, 2005). In direct treatment trials, there is evidence to suggest that individuals with refractory depression respond to antidepressant augmentation with L-methylfolate, and that higher doses (15 mg/d) are more effective than lower doses (1–5 mg/d) (Papakostas et al., 2012; Venkatasubramanian et al., 2013). There is evidence to suggest that *MTHFR* polymorphism status is associated with antidepressant treatment response, specifically in males receiving serotonin-norepinephrine re-uptake inhibitors (SNRIs) (Sun et al., 2013).

There appears to be a shared epigenetic vulnerability for schizophrenia, bipolar disorder (BPD) and MDD Peerbooms et al. (2011). Kevere et al. (2012) studied the association of serum homocysteine, severe mental illness and *MTHFR* polymorphisms. The highest serum levels of homocysteine were associated with the *MTHFR* 677TT genotype, and the most severely impaired individuals had cyclic episodes of psychosis with an affective component. Homocysteine levels were twice as high among heterozygotes who developed schizophrenia, and the course of their illness was more severe with recurring episodes and more affective symptoms (Kevere et al., 2014). Some studies have indicated that abnormal serum levels of vitamin B12 and folate, associated with cognitive impairments, psychotic and mood symptoms, were unrelated to *MTHFR* 677TT status (Moorthy et al., 2012).

The other polymorphism is the *MTHFR* 1298A > C form; it does not show as much ethnic or geographical variance. It is a less efficient form of the enzyme. In its homozygous CC form, 1298A > C is associated with twice the prevalence of major depression in a Slovak population (Evinova et al., 2012). There is also an association with bipolar disorder Peerbooms et al. (2011) and schizophrenia (Lajin et al., 2012). The 1298A > C genotype (but not the 677C > T genotype) may be associated with increased rates of ADD/ADHD (Gokcen et al., 2011; Baykal et al., 2019). Some studies indicate an association between the MTHFR gene mutations and increased risk of autism spectrum disorder. Case-control comparisons revealed significantly higher frequency of homozygosity as well as heterozygosity for both the 677C > T and 1298A > C genotypes among autistic versus non-autistic children (Liu et al., 2011; Rai, 2016). Also, migraine headache is associated with *MTHFR* polymorphisms. The 677TT polymorphism is more frequent among those with migraines, but the 1298CC polymorphism is more frequent among those with tension headaches and migraine with aura (Liu et al., 2019). Migraine has also been associated with higher homocysteine levels (Pizza et al., 2012; Rainero et al., 2013).

**FIGURE 6:**
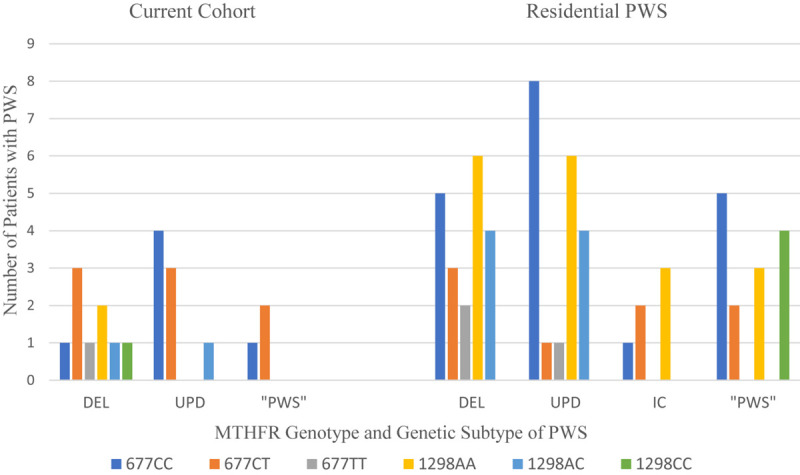
Distribution of MTHFR 677C > T and 1298A > C polymorphisms among PWS genetic subtypes from current cohort compared to residential PWS (Weisensel et al., 2016). DEL, deletion; UPD, uniparental disomy; IC, imprinting center defect; “PWS,” methylation positive, subtype undetermined.

Weisensel et al. (2016) studied 30 patients with PWS residing in a specialized residential setting (Residential PWS). There was no statistical relationship between *MTHFR* 677C > T or 1298A > C polymorphisms, PWS genetic subtype, psychiatric diagnosis, or the severity of mental illness as determined by a combination of the frequency of behavioral outbursts and the number of psychiatric medications needed to stabilize. Serum folate was within normal limits in all but one person who had a mild elevation at 35.1 (7–31.4) and who also had the 677CT/1298AA genotype. Homocysteine was marginally elevated 15.19 (5–15) in one person with 677CT/1298AA. Vitamin B12 levels were elevated in one third of the cohort and were higher among PWS deletion subtype with 677CT and 677TT alleles. The data from our cohort of PWS persons is limited to fifteen cases for the 677C > T polymorphism and 5 cases with the 1298A > C polymorphism. Figure 6 displays the data from our cohort with the data from the Residential PWS study by Weisensel et al. (2016).

In Table 8 the results from the current cohort and the Residential PWS study are compared with the frequencies of 677C > T and 1298A > C polymorphisms in a typical Caucasian population residing in the US, as reviewed in Nefic et al. (2018). Over half of our PWS cohort of referred patients had the intermediate metabolizer phenotype of *MTHFR* 677CT, which exceeds frequencies for both the Residential PWS and US Caucasian population. Another 20% have the poor metabolizer phenotype of 1298CC, which exceeds population norms. Unfortunately, our small sample size limits statistical analysis.

**TABLE 8:** MTHFR genotype frequency of 677C > T and 1298A > C gene polymorphisms in the current cohort, US Residential PWS, and US Caucasian population displayed by group and within group n/%.

	***MTHFR 677C > T***				***MTHFR 1298A > C***			
**Cohort**	***N***	**677CC**	**677CT**	**677TT**	***N***	**1298AA**	**1298AC**	**1298CC**
US Caucasian^†^	564	236/41.8%	246/43.6%	82/14.5%	574	265/46.2%	250/43.6%	59/10.3%
US Residential PWS*	30	19/63.3%	8/26.7%	3/10%	30	18/60%	8/26.7%	4/13.3%
Current: ALL PWS	15	6/40%	8/53.3%	1/6.7%	5	2/40%	2/40%	1/20%

^†^Adapted from Nefic et al. (2018). *Weisensel et al. (2016).

Compound genotypes have a clinically additive effect across several medical and psychiatric conditions. The frequency of compound genotypes of 677C > T and 1298A > C found in the current cohort is compared with the Residential PWS data and published norms (Ogino and Wilson, 2003) in Table 9.

**TABLE 9:** Frequency of compound genotypes of MTHFR 677C > T and 1298A > C in typical US population^*a*^ residential PWS^*b*^ and the current cohort^*c*^.

	**Compound Genotypes MTHFR 677C > T/MTHFR 1298A > C**								
***N***	**CC/AA**	**CC/AC**	**CC/CC**	**CT/AA**	**CT/AC**	**CT/CC**	**TT/AA**	**TT/AC**	**TT/CC**
7389^*a*^	15.6%	22.7%	9.1%	20.8%	20.3%	0.15%	11.2%	0.19%	0%
30^*b*^	26.6%	23.3%	13.3%	23.3%	3.3%	0	10%	0	0
5^*c*^	0	1	0	1	1	1	1	0	0

*^*a*^Typical US population (Ogino and Wilson, 2003). ^*b*^Residential PWS (Weisensel et al., 2016). ^*c*^Current PWS cohort.*

Data from Weisensel et al. (2016) indicates greater numbers of the following compound genotypes in Residential PWS compared to the typical population: CC/AA, CC/CC, and CT/AA. The frequency of CC/AC is comparable to the typical population, and the frequencies of CT/AC and TT/AA are less than expected. Analysis of the data from the Residential PWS study did not find any correlations between psychiatric diagnosis and single mutations in *MTHFR*, but the frequency of compound phenotypes among those with a diagnosis of psychosis was not ascertained. There is an increased incidence of psychosis in PWS (Sinnema et al., 2011; Manzardo et al., 2018) and association between compound genotypes and schizophrenia has been identified (Sazci et al., 2005; Foroughmand et al., 2015). Sazci et al. (2005) found that the risk of schizophrenia in Turkish male patients compared to healthy controls was associated with the 677TT and 1298CC genotypes and the 677TT/1298AA and 677CC/1298CC compound genotypes. Comparing patients with controls, the risk of schizophrenia in Iran was found to be associated with the following compound genotypes in descending frequency: 677CC/1298CC > 677TT/1298AA > 677CT/1298AA > 677CT/1298CC > 677CT/1298AC (Foroughmand et al., 2015). Compared to the typical US population, the Residential PWS cohort displayed increased frequencies of two of the at-risk compound genotypes (CC/CC and CT/AA) identified in the Middle Eastern studies of susceptibility to schizophrenia.

Other studies have explored the frequency of *MTHFR* compound genotypes among those who had ischemic or hemorrhagic embolism (Sazci et al., 2006). Among typical persons with venous thromboembolism (VTE), Liu et al. (2017) determined that twice as many had *MTHFR* compound genotypes compared to single mutations of *MTHFR*, and this finding was not associated with elevated serum homocysteine levels. This is of relevance to PWS as there is an increased risk of VTE (Butler et al., 2020).

MTHFR deficiency predisposes to major psychiatric disorders and life-threatening medical conditions. It is accepted clinically that supplementation with L-methyl folate is indicated in typical patients who have treatment resistant depression (Zajecka et al., 2016; Jain et al., 2019) and bipolar disorder (Nierenberg et al., 2017). Further, mood stabilizers such as lithium, valproic acid and lamotrigine may interfere with MTHFR function over time, and supplementation with oral L-methyl folate is recommended (Coppen and Bolander-Gouaille, 2005; Baek et al., 2013). Given the frequency of mood disorders in the PWS population, and the fact that more than half of individuals tested have the intermediate or poor phenotype, MTHFR testing or an empirical trial of the supplement L-methyl folate is recommended, especially in patients whose symptoms have been refractory to treatment.

### Human Leucocytic Antigens: HLA-A and HLA-B

One of the best recognized pharmacodynamic tests are alleles for the human leucocyte antigens (*HLA-A* and *HLA-B*) that determine cytotoxic T-cell function in the immune system. These surface receptors are heterodimers consisting of a major protein with multiple polymorphisms located on Ch6p21.3 and a minor invariant microglobulin located on Ch15q22, outside the PWS critical region of Ch15q11-q13. HLA-B^∗^1502 determines risk for Stevens-Johnson rash or toxic epidermal necrolysis. This severe, life threatening skin reaction is associated with the use of carbamazepine, lamotrigine, phenytoin, olanzapine, modafinil, and allopurinol in some individuals. This HLA test is now recognized and recommended by the FDA prior to starting carbamazepine, especially among Southeast Asian populations, where the odds ratio is reported to be 10 to 1 compared to near 0 for Europeans, Hispanics and Africans (Wang et al., 2017). Of the 33 persons with PWS referred for testing, only one of them carried the polymorphism for this cutaneous risk sensitivity, and this occurrence is compatible with statistical prediction of occurrence in the general population.

## Discussion

In this report, for the first time, the results of pharmacodynamic gene testing in a cohort of outpatients with PWS are presented with specific attention to the PWS genetic subtype. This combined cohort represents a referred population of patients with PWS whose pharmacogenomic testing was deemed medically necessary. In this clinically referred population, the number of patients with DEL was equal to UPD. The higher number of patients with UPD may be consistent with a referral bias due to the greater psychiatric co-morbidity among those who have this molecular subtype. Given the high likelihood of psychiatric co-morbidity in PWS with possible genetic subtype specificity, pharmacodynamic gene testing may be considered as an additional tool to inform psychotropic medication management.

When the results of pharmacodynamic testing for our cohort of 33 patients are pooled without regard to PWS genetic subtype, the distribution of most of the pharmacodynamic polymorphisms is comparable to population norms. However, differences were noted for PWS genetic subtypes for the polymorphism of the serotonin transporter promotor (increased frequency of the L allele in UPD) and *COMT* (increased frequency of VAL/VAL polymorphism in UPD and increased MET/MET in DEL). Serotonin 2A receptor gene testing revealed increased distribution of alleles in DEL with increased risk for side effects as well as diminished efficacy. Epigenetic effects on the efficiency of the serotonin 2C receptor in PWS are not expected to have genetic subtype specificity, and gene testing on 5 patients indicated the presence of the at-risk allele contributing to side effects. For *ADRA2A*, the frequency of the G allele is increased among the DEL subgroup, which may correlate with both treatment efficacy and adverse events, and there is an increase in the C allele among the UPD subgroup that may contribute to lesser efficacy of alpha-adrenergic agonists in treatment trials. MTHFR results indicate that both PWS genetic subtypes have an increased frequency of diminished function alleles, as well as increased frequency of compound genotypes associated with the risk of developing psychosis. Although limited by a small number in this clinical case series, polymorphisms of pharmacodynamic genes associated with PWS genetic subtype may contribute to disparities in treatment response and emergence of adverse effects. Further investigation of pharmacodynamic gene-gene interactions in the PWS population is recommended in a larger cohort.

Although there is no reason to suspect a difference in the frequency of serotonin transporter polymorphisms in PWS, there is a predominance of the L/L genotype among our cohort of persons with UPD subtype. The L allele of the serotonin transporter is well recognized as conferring resilience to stress. Although we typically consider PWS persons with both deletion and UPD to be equally susceptible to stress, we find more severe psychopathology among those persons with UPD subtype. It is possible that these findings indicate that people with UPD and PWS appear to tolerate rising levels of stress until their coping strategies fail precipitously, and then they present with symptoms of severe mental illness. It is also recognized that higher serotonin transporter activity is associated with greater sensitivity to the use of SSRIs. This may make those with UPD more susceptible to mood and behavioral activation, which clinically may appear similar to affective psychosis.

Among persons with PWS, it is suspected that the function of the serotonin 2C receptor is faulty due to the absence of function of the snoRNA gene HBII 52 (SNORD 115), which is located in the critical region and results in the failure to transcribe the antisense protein essential for proper editing of serotonin 2C receptor (Kishore and Stamm, 2006; Kishore et al., 2010). Further, the difference in expression of mRNA from *5HTRA* (excitatory) and *5HTRC* (inhibitory) results in an imbalance of serotonergic action in the prefrontal lobe resulting in mood and behavioral activation, especially during the childhood and adolescent years (Lambe et al., 2011). This has been reported to occur in up to one-third of juveniles and adolescents with PWS treated with SSRIs and some atypical antipsychotic medications (Durette et al., 2012; Gourash et al., 2015). In the current case series, up to 18% of persons with PWS carry the at-risk *HTR2A* alleles associated with sleep apnea and symptoms of metabolic syndrome. Although only 5 cases were tested for polymorphisms of the serotonin 2C receptor, all of them carried the CC allele placing them at greater risk for weight gain and movement disorder when treated with atypical neuroleptics. Currently, there are no studies examining the incidence of side effects of psychotropic medications, especially the SSRIs and atypical neuroleptics, that are so widely used in the PWS population of all ages. For example, even though most people with PWS live in circumstances with dietary management and food control in the environment, some individuals receiving atypical neuroleptics display increased food seeking, weight gain, and increased abdominal girth. Going forward, it would be important to ascertain if *HTR2A* and *HTR2C* polymorphisms are contributing factors.

With respect to the adrenergic receptor gene, *ADRA2A*, nearly 50% of those in our PWS cohort carried the low activity allele. Although this is typical in comparison with population normative data, there was some selectivity toward the UPD genotype in our data. Decreased receptor affinity may result in decreased efficacy of alpha-2 adrenergic agonists, which are currently being used to manage ADHD, skin picking, and aggressive/disruptive behavior in children and adolescents with PWS (Singh et al., 2019b). There is an increased dose of G allele among the deletion group compared to normative population data, indicating a tendency toward increased vascular reactivity with stress.

In our cohort, the distribution of *COMT* polymorphisms appeared to display genotypic specificity, with a greater number of persons with PWS DEL having the low activity MET allele and a greater number of persons with PWS and UPD and having the high activity VAL allele. COMT has a major effect on dopamine activity in the prefrontal cortex and plays a role in the etiology of the affective-psychotic spectrum of disorders. The homozygous VAL/VAL genotype has the lowest dopamine level and greatest risk for psychosis (Schacht, 2016). Further, dopamine level in the PFC decreases with age. It is tempting to associate the higher frequency of VAL/VAL genotype in our small PWS cohort to the larger group of persons with PWS and UPD who are at increased risk for affective psychosis with age. A linear association between low dopamine and psychosis has been abandoned in favor of a “U-shaped” hypothesis, suggesting that either extreme of dopamine availability in the PFC is likely to result in significant psychopathology. This would help to explain the extreme stress sensitivity among people with PWS as well as the increased risk for psychosis in both deletion and UPD subtypes. With respect to the behavioral phenotype of PWS, diminished working memory, selective attention, and impaired cognitive flexibility are several executive dysfunctions associated with low dopamine levels in the PFC (Fallon et al., 2012). Of interest, *COMT* gene expression has also been implicated in the placebo response, which is widely known anecdotally to occur in persons with PWS (Hall et al., 2019).

The results of *MTHFR* testing in our PWS cohort did not inform any PWS subtype specificity, but the frequency of 677TT was elevated compared to Residential PWS or US Caucasian. About one-third of the combined PWS cohort had diminished *MTHFR* 677CT phenotypic activity. The presence of the T allele increases stress response and predisposes to depression (Lok et al., 2013). There is evidence to suggest that those individuals who have been diagnosed with mood disorder may benefit from supplementation with L-methyl folate. Further, given the increased frequency of use of mood stabilizers (lithium, valproate, lamotrigine) in PWS, and evidence that these medications can interfere with MTHFR function, L-methyl folate supplementation is recommended. Analysis of compound genotypes is consistent with studies indicating an increased risk for schizophrenia. Additional studies are needed to explore the occurrence of these at-risk compound genotypes among those persons with PWS who have been diagnosed with psychosis. A larger cohort of subjects may identify statistically significant differences.

Finally, it was beyond the scope of this report with its small sample size to consider issues of gene-gene interactions. However, it is not unreasonable to expect that gene interactions might be-contributing to the risk of co-morbid psychiatric disorder in the PWS population. This is especially true of the interaction between serotonin transporter and serotonin 2A receptor in predicting response to treatment of depression and anxiety disorders with SSRIs and SNRIs (Lohoff et al., 2013). Further, Peerbooms et al. (2012) found that the number of T alleles of *MTHFR* 677CT in combination with the MET/MET polymorphism of *COMT* increased the risk of psychosis in response to environmental stress. Fryar-Williams (2016) discussed the impact of MTHFR deficiency on deficits in the methylation cycle related to the etiology of psychosis. Rahimi et al. (2016) have identified a synergistic interaction between the VAL allele of *COMT* and the T allele of the *MTHFR* 677CT polymorphism, which increased the incidence of Bipolar I disorder by a factor of 2.58 (*p* < 003). On the other hand, Wang et al. (2015) found that the VAL/VAL genotype of COMT in combination with the *MTHFR* 677TT genotype had a protective effect on the development of Bipolar II disorder among Han Chinese. Larger scale studies of pharmacodynamic factors in PWS are recommended in the future with a larger sample size to further address these preliminary observations. Pharmacodynamic gene testing can inform our understanding of the PWS genetic phenotype, enhance our knowledge of medication efficacy in treating psychiatric co-morbidity, and improve our awareness of potential side effects in the clinical setting.

## Limitations

There were three recognized limitations of this case series. The small number of patients did not allow us to attain sufficient power to perform statistical analysis. Patient data were deidentified for HIPPA compliance, so factors of age, gender, race and ethnic origin were not available for association. Psychiatric and behavioral history informing past experience with psychotropic medications, including doses and adverse effects, was not detailed. Although all patients met criteria for medical necessity, their psychiatric diagnosis and psychotropic medication history were not available for clinical correlation of testing results. In this case series, the phenotypic activity of the pharmacodynamic genes tested was inferred from the genotype. There are other factors that impact pharmacodynamic gene expression, such as epigenetic effects related to stress and effects of pharmacological treatment. Further, the results of cytochrome P450 gene polymorphisms were not part of this clinical report, and interactions between pharmacokinetic and pharmacodynamic genotypes are necessary to inform the clinical phenotype for each patient. Finally, our testing results came from three different pharmacogenomic companies whose panels differed in some respects as expected for an emerging field of medical care.

## Data Availability Statement

The original contributions presented in the study are included in the article/supplementary material, further inquiries can be directed to the corresponding authors.

## Ethics Statement

Ethical review and approval were not required for this report. This article is a scholarly description of a clinical case series of patients with PWS whose guardians consented for them to receive pharmacogenomic testing as part of their medical care at one of three specialty programs across the United States. All pertinent private or protected health information was eliminated, except for the genetic subtype of PWS and the pharmacodynamic gene results.

## Author Contributions

JF designed the format for this report and drafted the original manuscript while JD and MB revised and added to the manuscript. JF, JD, and MB contributed data from PWS individuals for the case series. All the authors agreed to the final version of the manuscript.

## Conflict of Interest

The authors declare that the research was conducted in the absence of any commercial or financial relationships that could be construed as a potential conflict of interest.
